# IPSE, an abundant egg-secreted protein of the carcinogenic helminth *Schistosoma haematobium*, promotes proliferation of bladder cancer cells and angiogenesis

**DOI:** 10.1186/s13027-020-00331-6

**Published:** 2020-10-22

**Authors:** Evaristus C. Mbanefo, Chinwike Terry Agbo, Yuanlong Zhao, Olivia K. Lamanna, Kim H. Thai, Shannon E. Karinshak, Mohammad Afzal Khan, Chi-Ling Fu, Justin I. Odegaard, Irina V. Saltikova, Michael J. Smout, Luke F. Pennington, Mark R. Nicolls, Theodore S. Jardetzky, Alex Loukas, Paul J. Brindley, Franco H. Falcone, Michael H. Hsieh

**Affiliations:** 1 Division of Urology, Department of Surgery, Children’s National Hospital, West Wing, 4th Floor, 111 Michigan Avenue NW, Washington, DC 20010 USA; 2 Carney Hospital, Boston, MA USA; 3 Mountainview Hospital, Las Vegas, NV USA; 4 Baylor Scott and White Health, Temple, TX USA; 5 Department of Microbiology, Immunology, and Tropical Medicine, School of Medicine & Health Sciences, The George Washington University, Washington, DC USA; 6 King Faisal Specialist Hospital and Research Center, Riyadh, Kingdom of Saudi Arabia; 7 Abbvie, Sunnyvale, CA USA; 8 Guardant Health, Redwood City, CA USA; 9 Siberian State Medical University, Tomsk, Russian Federation; 10 James Cook University, Townsville, Australia; 11 Department of Structural Biology, Stanford University, Stanford, CA USA; 12 Division of Pulmonology, Allergy, and Critical Care Medicine, Stanford University, Stanford, CA USA; 13 Institute of Parasitology, Justus-Liebig-Universität Gießen, Gießen, Germany

## Abstract

**Background:**

*Schistosoma haematobium,* the helminth causing urogenital schistosomiasis, is a known bladder carcinogen. Despite the causal link between *S. haematobium* and bladder cancer, the underlying mechanisms are poorly understood. *S. haematobium* oviposition in the bladder is associated with angiogenesis and urothelial hyperplasia. These changes may be pre-carcinogenic events in the bladder. We hypothesized that the Interleukin-4-inducing principle of *Schistosoma mansoni* eggs (IPSE), an *S. haematobium* egg-secreted “infiltrin” protein that enters host cell nuclei to alter cellular activity, is sufficient to induce angiogenesis and urothelial hyperplasia. Methods: Mouse bladders injected with *S. haematobium* eggs were analyzed via microscopy for angiogenesis and urothelial hyperplasia. Endothelial and urothelial cell lines were incubated with recombinant IPSE protein or an IPSE mutant protein that lacks the native nuclear localization sequence (NLS-) and proliferation measured using CFSE staining and real-time monitoring of cell growth. IPSE’s effects on urothelial cell cycle status was assayed through propidium iodide staining. Endothelial and urothelial cell uptake of fluorophore-labeled IPSE was measured. Findings: Injection of *S. haematobium* eggs into the bladder triggers angiogenesis, enhances leakiness of bladder blood vessels, and drives urothelial hyperplasia. Wild type IPSE, but not NLS-, increases proliferation of endothelial and urothelial cells and skews urothelial cells towards S phase. Finally, IPSE is internalized by both endothelial and urothelial cells. Interpretation: IPSE drives endothelial and urothelial proliferation, which may depend on internalization of the molecule. The urothelial effects of IPSE depend upon its NLS. Thus, IPSE is a candidate pro-carcinogenic molecule of *S. haematobium.*

**Summary:**

*Schistosoma haematobium* acts as a bladder carcinogen through unclear mechanisms. The *S. haematobium* homolog of IPSE, a secreted schistosome egg immunomodulatory molecule, enhances angiogenesis and urothelial proliferation, hallmarks of pre-carcinogenesis, suggesting IPSE is a key pro-oncogenic molecule of *S. haematobium.*

## Introduction

Urogenital schistosomiasis (UGS), primarily infection by the blood fluke, *Schistosoma haematobium*, affects the bladder and other pelvic organs. Egg deposition by the adult stage of the parasite in the bladder during infection is notable for its association with hematuria and bladder angiogenesis [1]. This suggests that this parasitic worm triggers aberrant host endothelial responses. *S. haematobium* oviposition is also linked to urothelial alterations such as hyperplasia [1–6]. It is unknown, however, if other factors produced by the *S. haematobium* adult worms, which live in the pelvic veins, also contribute to the bladder endothelial and urothelial changes of UGS. Both abnormal angiogenesis and epithelial hyperplasia have been associated with pre-carcinogenic changes in endodermal organs. Indeed, UGS is categorized as a group 1 carcinogen, i.e., deemed to cause cancer in humans, by the International Agency for Research on Cancer [7]. It is, however, unclear which components of *S. haematobium* eggs are pro-oncogenic.

One major protein secreted by the egg of *S. haematobium* is the ortholog of interleukin-4-inducing principle (IPSE) of the egg of the congener, *Schistosoma mansoni*, in which it was first discovered [8]. *S. mansoni* IPSE features numerous host modulatory properties. As indicated by its name, IPSE leads to secretion of IL-4 from basophils and mast cells by engaging IgE bound to the high affinity IgE receptor on these two cell types. IPSE also contains a nuclear localization sequence which guides the protein into the nuclei of host cells and presumably alters cellular activity [9, 10]. We have demonstrated that H03-H-IPSE, one of the major *S. haematobium* orthologs of IPSE [11], induces proliferation of mouse urothelial cells in vitro in a nuclear localization sequence-dependent manner [12]. Furthermore, H06-H-IPSE, another major *S. haematobium* ortholog of IPSE, is internalized by both urothelial and neuronal cells [13], indicating that IPSE may be taken up by and influence diverse cell types. This led us to hypothesize that IPSE drives proliferation of human urothelial cells, skews them towards S-phase of the cell cycle, and also induces angiogenesis.

Here we demonstrate that *S. haematobium* eggs, in the absence of *S. haematobium* adult stage worms, are sufficient to initiate the bladder endothelial and urothelial alterations of urogenital schistosomiasis. Furthermore, we show in vitro that H03-H-IPSE is taken up by both endothelial and urothelial cells. H03-H-IPSE triggers angiogenic behavior in endothelial cells in culture and orchestrates urothelial proliferation and S-phase cell cycle skewing in a nuclear localization sequence-dependent fashion.

## Materials and methods

### Mice

Female 6- to 7-wk-old BALB/c mice (Charles River Laboratories, Wilmington, MA, USA) were housed under 12 h light- dark cycles in temperature-controlled holding rooms with unlimited access to dry mouse chow and water. Newly received mice were acclimated to the animal facility for at least 1 week prior to experimental use.

### IPSE protein production and labeling

Recombinant H03 H-IPSE, H06 H-IPSE, and an NLS mutant of H03 H-IPSE (the wild type NLS sequence SKRRRKY changed to SAAGAAY) were produced in HEK293-6E cells, ﻿and then purified via immobilized-metal affinity chromatography using an 8x His tag in the construct as described [11]. H03 H-IPSE was conjugated to Alexa Fluor 488 using a Alexa Fluor 488 antibody labeling kit (Thermofisher Scientific, Waltham, MA) according to the manufacturer’s instructions; however, the pH was kept at 7.4 throughout the reaction to enrich for labeling of the terminal amine (pKa of 7.4). The efficiency of conjugation was confirmed by Nanodrop. The typical level of labeling was one mole of dye per mole of IPSE, which suggested IPSE was only labeled on the terminal amine. Avoiding labelling of lysine or arginine residues in the protein backbone is critical, as each of the positively charged amino acids in the NLS is essential for nuclear translocation [9]. Low labeling efficiency thus minimized the potential interference of the dye with IPSE’s functional domains.

### *S. haematobium* egg injection

Bladder wall injections were performed as described previously [14]. Female BALB/c mice were anesthetized with isoflurane, a midline lower abdominal incision was made, and the bladder was exteriorized. *S. haematobium* eggs (3000 eggs in 50 μl) were injected into the wall of the bladder. Abdominal incisions were subsequently closed with 4–0 Vicryl sutures, and the surgical site was treated once with topical antibiotic ointment.

### Histology

Bladder tissues were fixed in neutral buffered formalin, dehydrated, and embedded in paraffin. Five-micrometer sections were stained with hematoxylin and eosin. Histology was analyzed by a board-certified pathologist (JIO) in a blinded fashion.

### Ex vivo angiogenesis and microvascular leakage microscopy

Microvascular leakage was assessed 3 weeks following *S. haematobium* egg or vehicle injection using an established protocol [15]. This protocol involves the use of FITC-lectin to gauge vessels that are being perfused while also providing anatomic detail. Briefly, following FITC-lectin injection, 100 μL R50 Fluoro-Max red fluorescent microspheres, 0.048 μm in diameter (Thermo Scientific), were injected through the inferior vena cava. After 3–5 min in circulation, a sternotomy was performed, and the aorta was cannulated via the left ventricle with an 18-gauge angiocatheter and perfused with 1% paraformaldehyde for 3–5 min using a mini pump (Fisher Scientific). Grafts were harvested and mounted as described above. Microvascular permeability was assessed by using confocal microscopy to determine the extent of microsphere extravasation.

### Proliferation xCELLigence assay

Cells were seeded at 5000 cells per well in 200 μl of complete media in E-plates (ACEA Biosciences, San Diego, CA, USA) and grown overnight while monitored with an xCELLigence DP system (ACEA Biosciences) which monitors cellular events in real time by measuring electrical impedance across interdigitated gold micro-electrodes integrated on the bottom of tissue culture plates [16, 17]. Cells were washed three times with PBS and cultured with 180 μl EGM-2 basal media (no growth factors or supplements) and incubated for a minimum of 6 h before further treatment. Treatments were prepared at 10 × concentrations and added to each well in a total volume of 20 μl. The xCELLigence DP recorded cell index readings every 15 min for 3 days after treatment. Cell index readings were normalized before treatment and cell proliferation ratios were determined from four biological replicates and represent the relative numbers of cells compared to control cells. A two-way ANOVA with Holm–Sidak’s multiple comparisons test was used to compare IPSE treatment to medium-alone control, with *P* ≤ 0.05 deemed significant.

#### Cell lines

3B-11 and EOMA cells were obtained from ATCC (Manassas, Virginia). ATCC uses morphology, karyotyping, and PCR based approaches to confirm the identity of human cell lines and to rule out both intra- and interspecies contamination. These include an assay to detect species specific variants of the cytochrome C oxidase I gene (COI analysis) to rule out inter-species contamination and short tandem repeat (STR) profiling to distinguish between individual human cell lines and rule out intra-species contamination. HCV-29 cells were obtained from Dra. Monica Botelho. These cells have undergone STR profiling using the following markers: amelogenin, D8S1179, D18S51, D21S11, FGA, TH01, and vWA. The 3B-11, EOMA, and HCV-29 cells were Mycoplasma-free as established using the Lookout Mycoplasma PCR detection kit (Sigma-Aldrich).

#### Tubule formation assay

Growth factor-reduced Matrigel (Corning, Corning, NY, USA) was plated into a 96-well μ-angiogenesis plate (ibidi, Planegg, Germany) at 10 μl/well, and incubated at 37 °C in 5% CO_2_ in air for 60 min as described [18]. 3B-11 cells were detached using Trypsin/EDTA and resuspended in DMEM (Gibco), and seeded at 30,000 cells/well in medium supplemented with 10 μM sulforaphane (SFPH, Sigma) (negative control), or 1.8 μg/mL or 3.6 μg/mL IPSE. The ibidi plate was incubated for 5 h in a humidified atmosphere of 5% CO_2_ in air at 37 °C in a microscope stage top incubator (OKOLAB, Pozzuoli, Naples, Italy). At intervals, photomicrographs of cells and nascent and developed tubules were collected using a Leica DMi8 automated platform microscope under bright field at 2.5 × magnification, and LASX software (Leica).

#### Analysis of tubule formation

Automated angiogenesis assessment was performed on TFA 490^2^ pixel images by ImageJ (NIH) with the phase-contrast Angiogenesis Analyzer plugin tool as described [19, 20]. Settings used were as follows: 10 pixel minimum object size; 25 pixel minimum branch size; 2500 pixel artefactual loop size; 25 pixel isolated element size threshold; 30 pixel master segment size threshold; with iteration number of 3. The four output metrics (mesh count, segment count, segment length, and junction count) were either plotted directly or as a percentage relative to the medium-alone blank treatment (treatment measure divided by medium-alone measure). A two-way ANOVA with Holm–Sidak’s multiple comparisons test was used to compare IPSE treatment against medium-alone blank control for the four metrics with *P* ≤ 0.05 deemed significant.

Combining the four metrics into a single evenly weighted variable was accomplished through the calculation of *Z* standardized scores that were based on population values [21]. The formula below generates the *Z* score and represented the distance between the raw score and the population mean in units of the SD. Population values were estimated from 39 treatment replicates.

The combined robust *Z* score (Z*) was generated for each replicate from the median *Z* score of the four metrics. Z* scores were plotted, and IPSE treatments compared to medium-alone blank control using one-way ANOVA with Holm–Sidak’s multiple comparisons test, *P* ≤ 0.05 was considered to be statistically significant.

### Cell cycle analyses and CFSE assay

The human bladder epithelium (urothelium) cell line HCV-29 was grown in T-75 tissue culture flasks in complete DMEM media (Gibco) under 5% CO_2_ at 37 °C. For cell cycle assays, 1 × 10^5^ urothelial cells were co-incubated with IPSE. Following 48 h of culture, the cells were fixed and stained with propidium iodide for cell cycle analysis. For CFSE assays to assess cell proliferation, cells were stained with the CFSE dye prior to stimulation with IPSE and cultured for 48 h. The CFSE dye was evaluated post-culture by flow cytometry using the FITC channel. The intensity of CFSE dye, which halves with each cell cycle, was used to track the generations of urothelial cells.

### Endocytosis assays

HCV-29 human derived urothelial cells [see [22]] were grown in MEM (Thermo Fisher Scientific, Waltham, MA) with 10% fetal bovine serum (Sigma-Aldrich, St. Louis, MO). For internalization assays, floating cells and adherent cells (released via 0.12% trypsin (Sigma-Aldrich, St. Louis, MO) without EDTA) were washed in fresh medium, and aliquoted into 24 well plates at 200,000 cells/mL in 1 mL. The cells were incubated with Alexa 488-labeled H03 at 1 μg/mL (H03 was conjugated to Alexa 488 using a kit from Thermofisher Scientific, Waltham, MA) for 16 h at 37 °C. Cells were released via 0.12% trypsin without EDTA and washed 3 times with PBS (Sigma-Aldrich, St. Louis, MO). 0.4% trypan blue (Thermofisher Scientific, Waltham, MA) was added to the cells (1:4) to quench extracellular Alexa 488 signal. The cells were analyzed by flow cytometry (Beckman Coulter, CytoFLEX) to isolate the intracellular Alexa 488 signal. Data were analyzed using FlowJo and GraphPad.

### Statistical analysis

Statistical analyses were performed using GraphPad software. Except where noted otherwise, the Mann-Whitney U test and Student’s *t* test were used to evaluate statistical significance for nonparametrically and parametrically distributed data, respectively. *P* values of < 0.05 were defined as significant.

## Results

### *S. haematobium* eggs trigger urothelial hyperplasia of the bladder

**Fig. 1:**
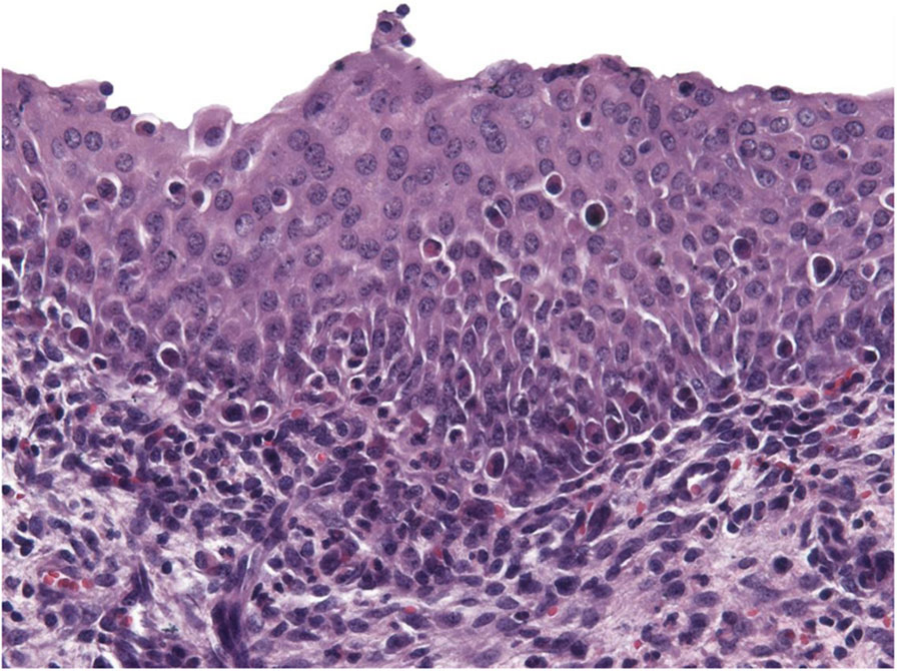
*Schistosoma haematobium* eggs are sufficient to induce urothelial hyperplasia in the bladder. Mice underwent bladder wall injection with *S. haematobium* eggs. Five weeks later, their bladders were harvested, processed, sectioned, and stained with hematoxylin and eosin. Image shows the typical hyperplastic urothelium (more than 3 cell layers thick) resulting from egg injection

We sought to determine whether *S. haematobium* eggs alone, in the absence of *S. haematobium* adult worms, were sufficient to induce urothelial hyperplasia in the bladder seen during urogenital schistosomiasis. To address the question, we injected *S. haematobium* eggs into the mouse bladder wall. Five weeks following egg injection, the urothelial lining of the bladder still exhibited significant hyperplasia (up to 12-cell thick urothelium compared to three cells characteristic of quiescent mouse bladder, Fig. 1). Thus, *S. haematobium* eggs were sufficient to trigger bladder urothelial hyperplasia.

### *S. haematobium* eggs initiate proliferation of bladder blood vessels with increased permeability

**Fig. 2:**
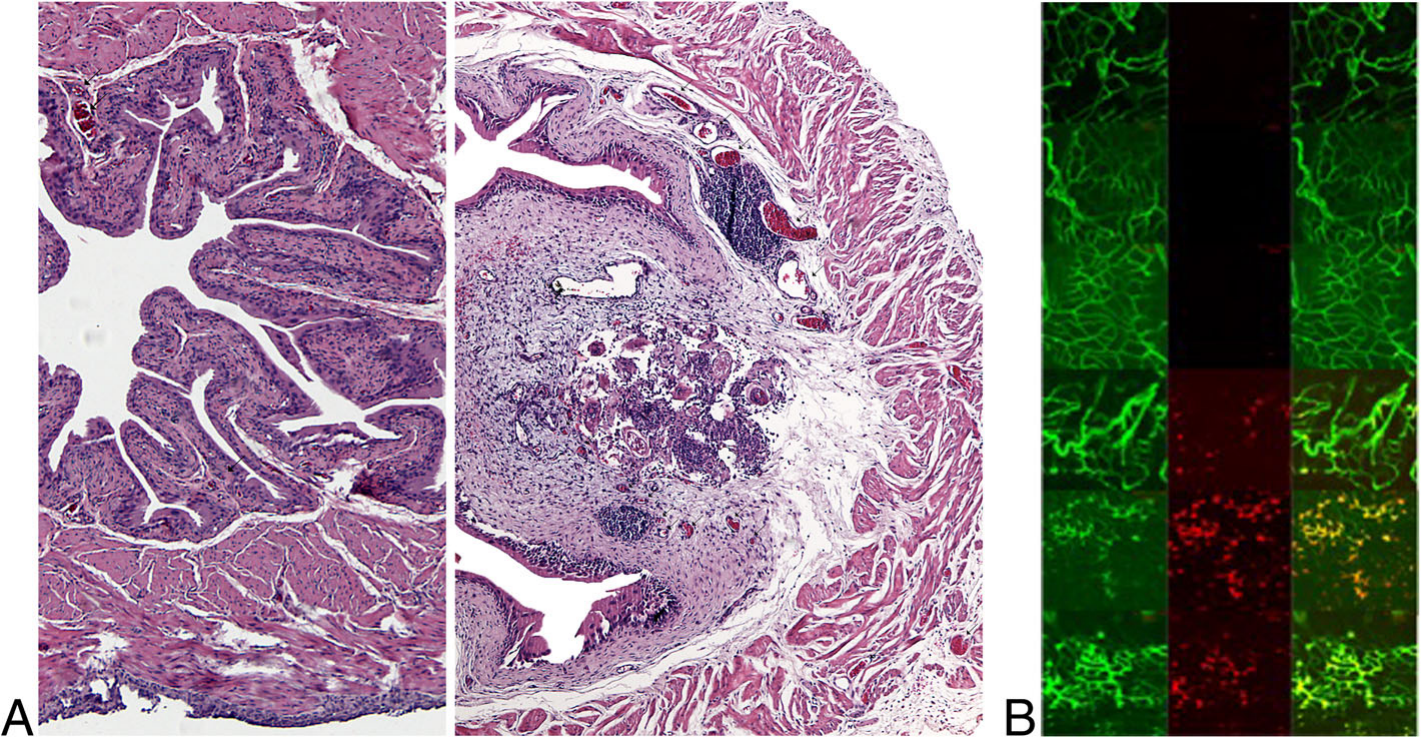
*Schistosoma haematobium* eggs are sufficient to induce angiogenesis and increased vascularpermeability in the bladder. **a**, Mice underwent bladder wall injection with S. *haematobium* eggs. Four days later their bladders were harvested, processed, sectioned, and stained with hematoxylin and eosin. Left and right panels show bladders injected with controlvehicle or *S. haematobium* eggs, respectively. Arrowheads indicate erythrocyte-containingblood vessels, which are more numerous in egg- versus vehicle-injected bladders. **b**, Miceunderwent bladder wall injection with *S. haematobium* eggs. Three weeks later mice were administered FITC-lectin to label blood vessels and Red Fluoro-Max microbeads (to measure vascular permeability) and their bladders harvested and examined by confocal microscopy. Left, middle, and right columns show FITC channel, red channel, and merged channels, respectively. Detection of red signal indicates leakage of Red Fluoro-Max microbeads out of blood vessels. Each row consists of representative images from a single mouse (*n* = 3 controls, *n* = 3 egg-injected)

Hematuria is a hallmark sign of urogenital schistosomiasis (UGS). By definition, hematuria involves leakage of erythrocytes from the lumen of blood vessels into surrounding bladder tissues and the bladder lumen. UGS is also associated with angiogenic changes in the bladder. We tested whether *S. haematobium* eggs were sufficient, without the presence of *S. haematobium* worms, to initiate angiogenesis and increase vascular permeability. Bladder wall injection of mice with *S. haematobium* eggs induced angiogenesis (Fig. 2a) featuring dilated, leaky blood vessels (Fig. 2b). We conclude that *S. haematobium* eggs suffice to orchestrate bladder angiogenesis and vascular leakiness.

### IPSE is internalized by endothelial and urothelial cells

**Fig. 3:**
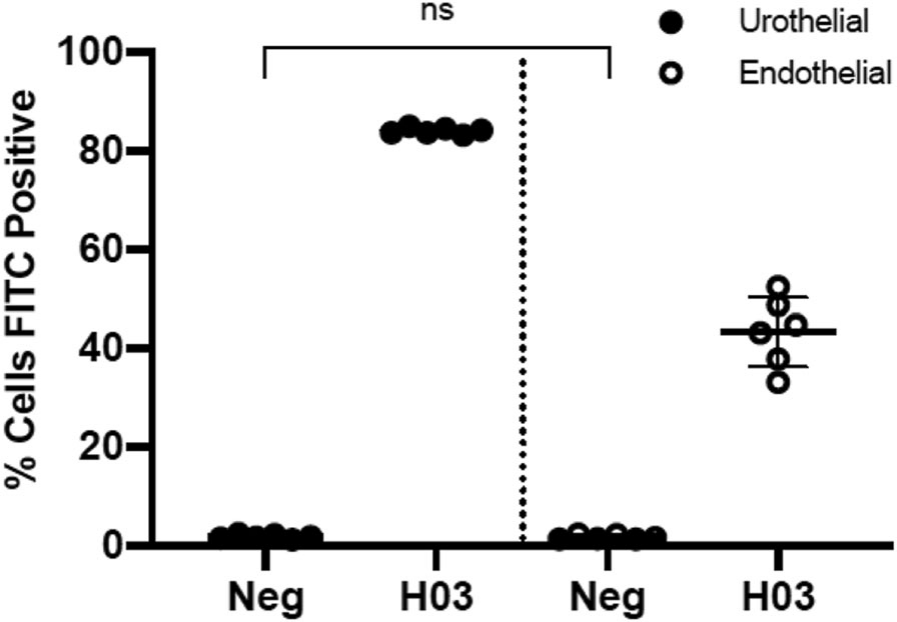
H03-H-IPSE is internalized by endothelial and urothelial cells. Alexa 488-labeled H03-H-IPSE was incubated with HCV-29 urothelial and EOMA endothelial cells. Extracellular signal was quenched with trypan blue, and the remaining intracellular Alexa 488 signal measured by flow cytometry. Each symbol denotes an individual culture well replicate. Figure shows one of two representative experiments

We next assayed the capacity of HCV-29 urothelial and EOMA endothelial cells to take up H03-H-IPSE. Using Alexa 488-labeled H03-H-IPSE, we noted that both cell types readily uptake the protein (Fig. 3).

### IPSE induces tubule formation by endothelial cells

Having demonstrated that whole *S. haematobium* eggs induce angiogenesis in vivo*,* we next sought to determine if recombinant H03-H-IPSE protein alone could induce similar changes in endothelial cells in vitro*.* In vitro tubule formation by the mouse 3B-11 endothelial cell line was enhanced by addition of 1.8 μg/mL or 3.6 μg/mL H03-H-IPSE to cultures (Additional file 1 and 2), indicating that this protein is pro-angiogenic (*p* < 0.05).

### IPSE mediates urothelial cell proliferation in a nuclear localization sequence-dependent manner

**Fig. 4:**
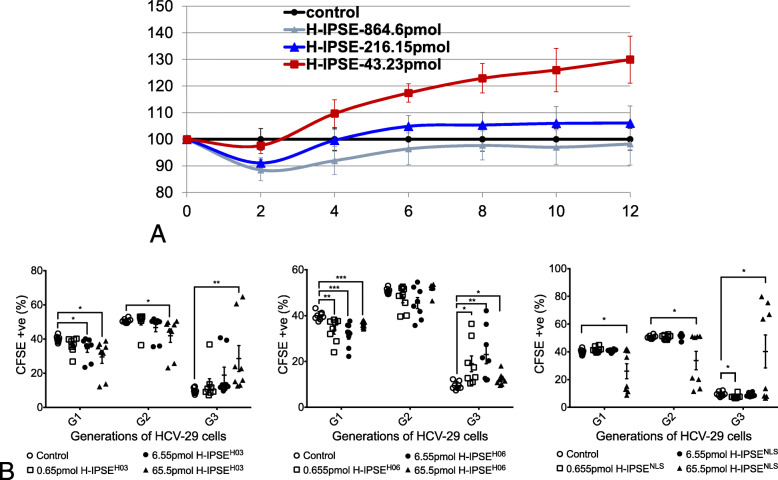
H-IPSE enhances urothelial cell proliferation in a nuclear localization sequence-dependent fashion. **a**, H03-H-IPSE increases urothelial cell proliferation. HCV-29 human urothelial cells were incubated with 0, 43, 216, or 864 pmol of H03-H-IPSE and electrical impedance (cellular proliferation) measured in real-time using the xCELLigence platform. **b**, left panel, CFSE-labeled HCV-29 human urothelial cells were incubated with up to 65 pmol of H03-H-IPSE and analyzed for evidence of proliferation (decreased CFSE content per cell with each successive generation of cells). Each symbol denotes an individual culture well replicate (*n* = 8 for each culture condition). B, middle panel, CFSE-labeled HCV-29 human urothelial cells were incubated with up to 65 pmol of H06-H-IPSE and analyzed for evidence of proliferation. Each symbol denotes an individual culture well replicate (*n* = 8 for each culture condition). B, right panel, CFSE-labeled HCV-29 human urothelial cells were incubated with up to 65 pmol of a nuclear localization mutant of H03-H-IPSE and analyzed for evidence of proliferation (decreased CFSE content per cell with each successive generation of cells). Each symbol denotes an individual culture well replicate

Given *S. haematobium* eggs stimulate urothelial hyperplasia in the bladder, we next assayed the ability of recombinant H03-H-IPSE protein to trigger the in vitro equivalent, proliferation of urothelial cells. Electrical impedance measurements of cellular proliferation using the xCELLigence platform revealed that incubation of HCV-29 human urothelial cells with 43 pmol, but not 216 or 864 pmol (possibly cytotoxicity), of H03-H-IPSE induced proliferation of the urothelial cells (Fig. 4a).

The pro-proliferative effect of H03-H-IPSE on urothelial cells was also verified using CFSE staining. These assays demonstrated that H03-H-IPSE, up to the tested concentration of 65 pmol, drove proliferation of HCV-29 cells in a concentration-dependent fashion (Fig. 4b). H06-H-IPSE also induced proliferation of HCV-29 cells. Incubation of HCV-29 cells with a nuclear localization sequence mutant of H03-H-IPSE failed to enhance proliferation, suggesting that H03-H-IPSE’s pro-proliferative influence depends upon the protein’s ability to migrate into the nucleus of the host cell.

### IPSE skews urothelial cell cycle status in a nuclear localization sequence-dependent fashion

**Fig. 5:**
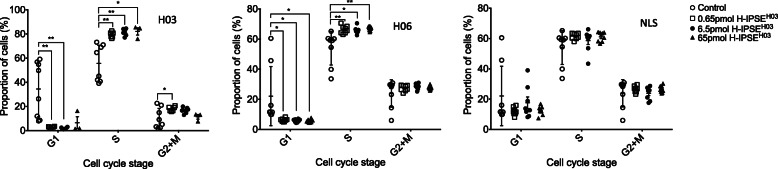
H-IPSE polarizes urothelial cell cycle status in a nuclear localization sequence-dependent manner. Left panel, propidium iodide-labeled HCV-29 human urothelial cells were incubated with up to 65 pmol of H03-H-IPSE and analyzed for cell cycle status. Each symbol denotes an individual culture well replicate (*n* = 8 for each culture condition). Middle panel, propidium iodide-labeled HCV-29 human urothelial cells were incubated with up to 65 pmol of H06-H-IPSE and analyzed for cell cycle status. Each symbol denotes an individual culture well replicate (*n* = 8 for each culture condition). Right panel, CFSE-labeled HCV-29 human urothelial cells were incubated with up to 65 pmol of a nuclear localization mutant of H03-H-IPSE and analyzed for cell cycle status. Each symbol denotes an individual culture well replicate

Our findings that H03-H-IPSE stimulated urothelial proliferation led us to examine whether this protein alters the cell cycle status of urothelial cells. Through propidium iodide staining of H03-H-IPSE-exposed HCV-29 urothelial cells, we ascertained that this protein indeed skews cells towards S-phase (Fig. 5). The ability of H03-H-IPSE to bias urothelial cells towards S-phase was nuclear localization sequence-dependent, since the nuclear localization sequence mutant form of H03-H-IPSE did not change the cell cycle status of HCV-29 cells.

## Discussion

Schistosomiasis affects more than 200 million people worldwide [23]. The majority of cases affect the urogenital tract, caused mostly by *S. haematobium.* As a blood fluke, *S. haematobium* adult worms live primarily in the pelvic veins, where worm pairs lay eggs that lodge in the pelvic organs. Eggs laid in the bladder induce granuloma formation, which is thought to facilitate passage of eggs into the urinary stream [24, 25]. Eggs voided into fresh bodies of water hatch into miracidia, which infect intermediate snail hosts. Infected snails release cercariae, the larval stage which infects humans.

The complex life cycle of *S. haematobium* depends upon the parasite successfully negotiating host tissue and immune responses that may threaten its survival. For instance, hematuria is a cardinal sign of urogenital schistosomiasis, and represents a compromised urothelial and endothelial barrier which allows *S. haematobium* to continue reproducing. However, unchecked damage to the urothelium and bladder blood vessels results in hemorrhage and even host death, which is counterproductive to any parasite [26]. Hence, it is possible that *S. haematobium* and humans have co-evolved survival strategies. The exuberant proliferative response of the urothelium and endothelium to *S. haematobium* eggs may be one such strategy, since this likely promotes bladder tissue repair.

Schistosomes may induce host tissue repair responses, in particular angiogenesis, to promote egg expulsion. Turner et al. reported that deposition of *S. mansoni* eggs in Peyers’ patches of the mouse small intestine is associated with vascular remodeling and an expanded venule network [27]. In mice deficient in Peyers’ patches, egg excretion is lessened, leading to more eggs entrapped in tissues, and consequently worsened host morbidity. We postulate that *S. haematobium* eggs similarly initiate angiogenesis in the bladder to facilitate their expulsion into the urinary stream.

Angiogenesis and urothelial proliferation benefit the *S. haematobium-*infected host in the short term but have been postulated to facilitate bladder carcinogenesis. *S. haematobium* is one of a handful of helminths that are known to be carcinogenic [28]. Angiogenesis, such as that seen in bladders containing *S. haematobium* eggs, is a crucially important process for tumors, since their rapid growth puts them at risk of outstripping their blood supply. Expansion of local vascular beds may also enhance hematogenous spread of metastases. Urogenital schistosomiasis is associated with urothelial hyperplasia, a potentially pre-cancerous feature in the bladder.

Despite the prominence of urothelial and endothelial aberrations in urogenital schistosomiasis, our comprehension of the underpinnings of these pathological processes is limited. One mystery is whether *S. haematobium* eggs, without adult worms, are sufficient to activate urothelial hyperplasia and angiogenesis in the bladder. This is a significant matter because *S. haematobium* worms can live for years within their human hosts, whereas parasite eggs can remain in the bladder wall for decades and continue to drive chronic inflammation, even after successful treatment of infection. Understanding the contributions of *S. haematobium* worms to bladder pathogenesis is important to improving our knowledge base of chronic urogenital schistosomiasis. To that end, we observed that when *S. haematobium* eggs are injected into the bladder walls of mice, this elicits significant urothelial hyperplasia, angiogenesis, and vascular leakiness. These bladder changes are not seen when mice are injected with control vehicle. These findings intimate that *S. haematobium* worms play no or minimal role in the pathological changes of the bladder associated with urogenital schistosomiasis. Finally, these findings point to one or more *S. haematobium* egg-associated factors which mediate urothelial and endothelial changes.

We hypothesized that one of these egg-associated factors was the Interleukin-4 inducing Principle of *Schistosoma mansoni* Eggs (IPSE) [29]. IPSE, also known as α-1 [8], features many host immunomodulatory functions. First, IPSE ligates Fcε receptor-bound IgE on the surface of mast cells and basophils to trigger IL-4 and IL-13 secretion [29–32]. Splenocytes from mice immunized with IPSE produce IFN-gamma, TNF, IL-4, IL-5, and IL-13 [33]. IPSE also binds to immunoglobulins on the surface of B regulatory cells (Bregs) and thereby induces IL-10 production by these cells [34]. IPSE can also sequester chemokines; it was previously known as *S. mansoni* chemokine-binding protein (smCKBP) [35]. Lastly, IPSE contains a nuclear localization sequence which directs the protein to host cell nuclei [9, 11], where it modulates transcription [10, 36].

The manifold immunomodulatory properties of IPSE may be particularly relevant given the co-endemicity of urogenital schistosomiasis with other infections that trigger significant cytokine responses, such as malaria and HIV. COVID-19-associated cytokine dysregulation may also be altered by IPSE released by *S. haematobium* eggs in co-infected individuals. These are important areas for future inquiry.

Although IPSE has multiple immunomodulatory properties, our in vitro work indicates that H03-H-IPSE mediates its urothelial and endothelial effects via non-immune mechanisms, including through its nuclear localization sequence. The similar uptake of H03-H-IPSE by endothelial and urothelial cells hints that endocytosis occurs in either a non-specific fashion, or that these cell types share a receptor for H03-H-IPSE. We speculate that once IPSE is internalized by host cells, it may trigger pro-carcinogenic programs. Indeed, Roderfeld et al. showed that IPSE activated hepatocellular carcinoma-associated proto-oncogenes, namely c-Jun and its associated signaling molecule, STAT3 [36]. This supports a potential role for H03-H-IPSE in promoting bladder oncogenesis.

Another interesting issue regarding the potential causal link between IPSE and schistosomal bladder carcinogenesis is whether this molecule promotes the high rates of squamous cell carcinoma seen in association with *S. haematobium* infection [37–40]. This remains an open question, since we did not have an immortalized schistosomiasis-associated squamous cell bladder carcinoma cell line available for testing. However, schistosomal bladder cancer can still be associated with carcinomas arising from the urothelium, the tissue of origin for the HCV-29 cells tested herein.

Superficially, targeting IPSE to prevent schistosomal bladder cancer appears to be a worthy prophylactic approach. However, it has been reported that immunization of *S. mansoni* mice with IPSE leads to larger granulomas with enhanced macrophage activity and a mixed type 1 and type 2 immune response [33]. Since granulomas contribute to host tissue fibrosis, neutralization of IPSE during schistosomiasis may actually be undesirable. Thus, a vaccine directed against IPSE may not ameliorate the pathology of schistosomiasis.

We speculate that given IPSE is an abundant egg-secreted protein, diagnostic assays designed to measure IPSE expression in *S. haematobium-*infected individuals may provide valuable morbidity assessment and prognostic information that is not available through current methods.

This investigation has noteworthy limitations. Although we hypothesize that H03-H-IPSE is sufficient to drive urothelial and endothelial proliferation and related effects, we have not demonstrated that it is necessary. There may be additional *S. haematobium* egg-derived factors which contribute to urothelial hyperplasia and angiogenesis during urogenital schistosomiasis. Ideally, transgenic approaches would be used to show that H03-H-IPSE is critical in angiogenesis and urothelial hyperplasia in vivo*.* This approach could be in reach for future studies given that both germline transgenesis and genome editing of egg-expressed genes have now been reported in other work [41, 42]. Another important caveat of our work is that the in vivo studies were performed using female mice. Men are at higher risk than women for development of all forms of bladder cancer (i.e., both schistosomiasis-related and -unrelated). Part of this risk is due to the importance of androgen receptor signaling in bladder carcinogenesis [43]. There are also reports of a possible relationship between estrogen-DNA adducts and schistosomal bladder oncogenesis [44]. In Honeycutt et al., we did not observe obvious differences in H&E-based histology of granulomatous inflammation in wild type male versus female mice [2]. We also did not observe histological differences when these wild type mice were administered tamoxifen versus control vehicle, suggesting that estrogen receptor-mediated signaling did not cause apparent differences. However, it is possible that sex and sex hormones influence other aspects of urogenital schistosomiasis that we have not yet studied.

In conclusion, we have provided data that support the hypothesis that *S. haematobium* eggs are sufficient to initiate bladder urothelial hyperplasia, angiogenesis, and vascular permeability associated with urogenital schistosomiasis. These changes may be orchestrated by IPSE. Our observations are particularly striking considering that IPSE can increase proliferation of transformed urothelial and endothelial cell lines that, at baseline, already exhibit significant proliferation. IPSE may indeed be a pro-oncogenic factor of *S. haematobium.*

## Supplementary information

**Additional file 1.** H03-H-IPSE drives increased tubule formation by 3B-11 endothelial cells. A, time-lapse movie showing spontaneous tubule formation by 3B-11 cells.

**Additional file 2 **H03-H-IPSE drives increased tubule formation by 3B-11 endothelial cells. B, time-lapse movie showing increased tubule formation by 3B-11 cells following incubation with H03-H-IPSE. C, Z-scores of spontaneous vs. IPSE-associated tubule formation by 3B-11 cells. 95% CI error bars shown, comparisons made against the control with one-way ANOVA and Dunnet multiple analysis correction. * *p*<0.05.

## Data Availability

All data and material are available upon reasonable request.

## References

[1] Botros SS (2008). Schistosoma haematobium (Egyptian strain): rate of development and effect of praziquantel treatment. J Parasitol.

[2] Honeycutt J (2015). Schistosoma haematobium egg-induced bladder urothelial abnormalities dependent on p53 are modulated by host sex. Exp Parasitol.

[3] Mbanefo EC (2020). Interleukin-4 signaling plays a major role in urogenital Schistosomiasis-associated bladder pathogenesis. Infect Immun.

[4] Santos J (2014). P53 and cancer-associated Sialylated Glycans are surrogate markers of Cancerization of the bladder associated with Schistosoma haematobium infection. PLoS Negl Trop Dis.

[5] Mostafa MH (1999). Relationship between Schistosomiasis and bladder cancer. Clin Microbiol Rev.

[6] Fu C-L (2012). A novel mouse model of Schistosoma haematobium egg-induced immunopathology. PLoS Pathog.

[7] IARC Working Group on the Evaluation of Carcinogenic Risks to Humans. Biological agents. Volume 100 B. A review of human carcinogens. IARC Monogr Eval Carcinog Risks Hum [Internet]. 2012;100:1–441. Available from: https://pubmed.ncbi.nlm.nih.gov/23189750/. [cited 2020 Oct 14]. PMC4781184 23189750

[8] Schramm G (2006). IPSE/alpha-1: a major immunogenic component secreted from Schistosoma mansoni eggs. Mol Biochem Parasitol.

[9] Kaur I (2011). Interleukin-4-inducing principle from Schistosoma mansoni eggs contains a functional C-terminal nuclear localization signal necessary for nuclear translocation in mammalian cells but not for its uptake. Infect Immun.

[10] Mbanefo EC (2019). IPSE, a urogenital parasite-derived immunomodulatory protein, ameliorates ifosfamide-induced hemorrhagic cystitis through downregulation of pro-inflammatory pathways. Sci Rep.

[11] Pennington LF (2017). H-IPSE is a pathogen-secreted host nucleus-infiltrating protein (Infiltrin) expressed exclusively by the Schistosoma haematobium egg stage. Infect Immun.

[12] Zee RS (2019). IPSE, a parasite-derived host immunomodulatory protein, is a potential therapeutic for hemorrhagic cystitis. Am J Physiol Physiol.

[13] Ishida K, et al. IPSE, a parasite-derived, host Immunomodulatory Infiltrin protein, alleviates Resiniferatoxin-induced bladder pain. bioRxiv. 2020. 10.1101/2020.06.11.146829. 10.1177/1744806920970099 PMC7756320 33342372

[14] Fu C-LL (2011). Mouse bladder wall injection. J Vis Exp.

[15] Khan M (2013). Targeting complement component 5a promotes vascular integrity and limits airway remodeling. Proc Natl Acad Sci U S A.

[16] Haugen B (2018). Granulin secreted by the food-borne liver fluke Opisthorchis viverrini promotes angiogenesis in human endothelial cells. Front Med.

[17] Ke N, et al. The xCELLigence system for real-time and label-free monitoring of cell viability. Methods Mol Biol. 2011. 10.1007/978-1-61779-108-6_6. 10.1007/978-1-61779-108-6_6 21468966

[18] Arnaoutova I, et al. In vitro angiogenesis: endothelial cell tube formation on gelled basement membrane extract. Nat Protoc. 2010. 10.1038/nprot.2010.6. 10.1038/nprot.2010.6 20224563

[19] DeCicco-Skinner KL, et al. Endothelial cell tube formation assay for the in vitro study of angiogenesis. J Vis Exp. 2014. 10.3791/51312. 10.3791/51312 PMC4540586 25225985

[20] Carpentier G, et al. Angiogenesis analyzer for ImageJ. 4th ImageJ User Dev. Conf. 2012. http://image.bio.methods.free.fr/ImageJ/?Angiogenesis-Analyzer-for-ImageJ.

[21] Zhang XD, et al. The use of strictly standardized mean difference for hit selection in primary RNA interference high-throughput screening experiments. J Biomol Screen. 2007. 10.1177/1087057107300646. 10.1177/1087057107300646 17435171

[22] Nacif-pimenta R (2019). Differential responses of epithelial cells from urinary and biliary tract to eggs of Schistosoma haematobium and S . Mansoni. Sci Rep.

[23] Utzinger J (2019). Schistosomiasis. Encyclopedia of environmental health.

[24] Hams E (2013). The schistosoma granuloma: friend or foe?. Front Immunol.

[25] Schwartz C (2018). Schistosoma “eggs-Iting” the host: granuloma formation and egg excretion. Front Immunol.

[26] Fu C (2014). Macrophages are required for host survival in experimental urogenital Schistosomiasis. FASEB J.

[27] Turner JD (2012). Blood flukes exploit Peyer’s patch lymphoid tissue to facilitate transmission from the mammalian host. PLoS Pathog.

[28] Honeycutt J (2014). Controversies and challenges in research on urogenital schistosomiasis-associated bladder cancer. Trends Parasitol.

[29] Schramm G (2003). Molecular characterization of an interleukin-4-inducing factor from Schistosoma mansoni eggs. J Biol Chem.

[30] Mbanefo EC (2018). Therapeutic exploitation of IPSE, a urogenital parasite-derived host modulatory protein, for chemotherapy-induced hemorrhagic cystitis. FASEB J.

[31] Knuhr K, Langhans K, Nyenhuis S, Viertmann K, Kildemoes AMO, Doenhoff MJ, et al. Schistosoma mansoni Egg-Released IPSE / alpha-1 Dampens Inflammatory Cytokine Responses via Basophil Interleukin ( IL ) -4 and IL-13. Front Immunol. 2018;9:1–15. 10.3389/fimmu.2018.02293 PMC6191518 30364177

[32] Meyer NH, et al. A crystallin fold in the interleukin-4-inducing principle of Schistosoma mansoni eggs (IPSE/alpha-1) mediates IgE binding for antigen-independent basophil activation. J Biol Chem. 2015. 10.1074/jbc.M115.675066. 10.1074/jbc.M115.675066 PMC4571962 26163514

[33] Fahel JS (2010). IPSE/alpha-1 of Schistosoma mansoni egg induces enlargement of granuloma but does not alter Th2 balance after infection. Parasite Immunol.

[34] Haeberlein S (2017). Schistosome egg antigens, including the glycoprotein IPSE/alpha-1, trigger the development of regulatory B cells. PLoS Pathog.

[35] Smith P (2005). Schistosoma mansoni secretes a chemokine binding protein with antiinflammatory activity. J Exp Med.

[36] Roderfeld M (2019). *Schistosoma mansoni* egg-secreted antigens activate hepatocellular carcinoma-associated transcription factors c-Jun and STAT3 in hamster and human hepatocytes. Hepatology.

[37] Thomas JE (1990). Relationship between bladder cancer incidence, Schistosoma haematobium infection, and geographical region in Zimbabwe. Trans R Soc Trop Med Hyg.

[38] Bhagwandeen SB (1976). Schistosomiasis and carcinoma of the bladder in Zambia. S Afr Med J.

[39] Groeneveld AE (1996). Bladder cancer in various population groups in the greater Durban area of KwaZulu-Natal, South Africa. Br J Urol.

[40] Salem S (2011). Successful control of schistosomiasis and the changing epidemiology of bladder cancer in Egypt. BJU Int.

[41] Rinaldi G (2012). Germline transgenesis and insertional mutagenesis in Schistosoma mansoni mediated by murine leukemia virus. PLoS Pathog.

[42] Ittiprasert W, et al. Programmed genome editing of the omega-1 ribonuclease of the blood fluke, *Schistosoma mansoni*. Elife. 2019. 10.7554/eLife.41337. 10.7554/eLife.41337 PMC6355194 30644357

[43] Miyamoto H (2007). Promotion of bladder cancer development and progression by androgen receptor signals. J Natl Cancer Inst.

[44] Botelho MC (2013). Tumour-like phenotypes in urothelial cells after exposure to antigens from eggs of Schistosoma haematobium: an oestrogen–DNA adducts mediated pathway?. Int J Parasitol.

